# Colonial breeding impacts potentially fitness-relevant cognitive processes in barn swallows

**DOI:** 10.1007/s10071-024-01841-1

**Published:** 2024-03-02

**Authors:** Angela Medina-García, Ellen Scherner, Molly T. McDermott, Mark E. Hauber, Rebecca J. Safran

**Affiliations:** 1 Department of Ecology and Evolutionary Biology, University of Colorado at Boulder, 334 UCB, 1900 Pleasant Street, Boulder, CO 80309 USA; 2 Advanced Science Research Center and Program in Psychology, Graduate Center of the City University of New York, 85 St. Nicholas Terrace, New York, NY 10031 USA

**Keywords:** Barn swallow, Cognition, Colony size, Fitness, Predator intrusions, Reproductive success, Social information

## Abstract

Many animals breed colonially, often in dense clusters, representing a complex social environment with cognitive demands that could ultimately impact individual fitness. However, the effects of social breeding on the evolution of cognitive processes remain largely unknown. We tested the hypothesis that facultative colonial breeding influences attention and decision-making. Barn swallows (*Hirundo rustica*) breed in solitary pairs or in a range of colony sizes, up to dozens of pairs. We tested for selective attention to social information with playbacks of conspecific alarm calls and for decision-making with simulated predator intrusions, across a range of colony sizes from 1 to 33 pairs. We also evaluated the adaptive value of both processes by measuring seasonal reproductive success. Swallows breeding in larger colonies were more selective in their attention to social information. Birds breeding in larger colonies were also less risk averse, deciding to return more quickly to their nests after a predator approach paradigm. Finally, birds that showed higher selective attention hatched more eggs and birds that returned to their nests more quickly after a predator intrusion had more nestlings. Although we cannot fully attribute these fitness outcomes to the cognitive measures considered in this study, our results suggest that social breeding plays a role in adaptively shaping both the acquisition of social information and decision-making.

**Supplementary Information:**

The online version contains supplementary material available at 10.1007/s10071-024-01841-1.

## Introduction

Cognition encompasses all of the sensory, perceptual, and neural mechanisms through which animals acquire, process, and retain information for decision-making (Shettleworth 2010). From foraging through predator avoidance to social behavior, cognitive processes underlie animal decisions. Despite the putative impacts of cognitive processes on individuals’ fitness, the factors shaping social cognition processes in natural populations, and their adaptive consequences, remain poorly understood (Morand-Ferron and Quinn 2015).

Many vertebrates breed colonially, often in dense clusters (Perrins and Birkhead 1984). Such social breeding systems are broadly distributed across taxa and, for instance, have evolved independently numerous times across avian lineages (Lack 1968; Rolland et al. 1998). The evolutionary origins and reproductive impacts of social breeding have been widely studied in birds (Møller 1987; Rolland et al. 1998; Safran et al. 2007; Serrano et al. 2005; Snapp 1976; Varela et al. 2007). However, the impacts of social breeding on the evolution of cognitive processes remain largely unexplored.

Colonial breeding involves frequent interactions with conspecifics, and demands high levels of information-acquisition and -processing, both key cognitive processes in survival and reproduction. The social intelligence hypothesis posits that competition and cooperation resulting from a social lifestyle have shaped cognitive processes, particularly those underlying the mediation of social relationships (Humphrey 1976; Jolly 1966; Seyfarth and Cheney 2015). Intraspecifically, this hypothesis predicts that individuals within the same species that experience a socially complex environment should also exhibit enhanced cognitive abilities. Although breeding colonies are not always defined by long-term social aggregations, many breeding adults return to the same breeding site year after year (Safran 2004; Shields 1984) and, thus, they can experience frequently repeated interactions among individuals both within and between breeding seasons. Therefore, the social intelligence hypothesis provides a highly relevant and fruitful framework for studying the impacts of social breeding on the evolution of cognitive processes. Individuals must continuously gather and keep track of information from their social environment to successfully reproduce and survive. Predatory or brood parasitic threat information, for instance, can be acquired either from individual experience (personal information) or from monitoring the actions of conspecifics or heterospecifics (social information) (Danchin et al. 2004; Lawson et al. 2020; Thorogood and Davies 2012).

Social environments impose specific cognitive demands, favoring not only the acquisition of social information, but also selective attention to relevant stimuli. In turn, for these cognitive processes to evolve by natural selection, the behavioral manifestations of these processes (i.e., measurable cognitive outputs) must have a direct impact on the individuals’ reproductive success. Here, we use the social intelligence hypothesis as a framework to test whether facultative colonial breeding systems have promoted the evolution of cognitive processes geared towards the adaptively selective acquisition of social information and subsequent decision-making.

This hypothesis proposes two testable predictions associated with cognitive traits relevant to social breeding:

A. *Selective attention to social information*: Individuals in larger colonies will be more selective in their attention to social information (i.e., less responsive to conspecific alarm calls) than individuals breeding in smaller colonies or solitarily. The rationale is that the relevance of social information for the focal individual is less in larger colonies due to the increased availability of this type of information in such colonies.
B. *Decision-making*: Individuals in larger colonies will be likely to decide to return to the nest more quickly (i.e., show shorter latency to return to the nest) after a predator intrusion than individuals breeding in smaller colonies or solitarily. The rationale is that adult birds from larger colonies may be at lower risk *per capita* and, therefore, show lower risk avoidance (despite the presence of a potential predator) and, in consequence, higher return rates. In other words, parents at larger colonies are at a lower risk from predators, and thus, more likely to return quickly to feed their nestlings compared to solitary breeders, which are more vulnerable to predators.

Studies that evaluate the impact of ecologically relevant cognitive processes on fitness in free-ranging animals are rare, but they are essential in order to establish the adaptive value of these processes in the context of social living. Here we also test whether cognitive processes such as selective attention to social information and decision-making are linked to measures of reproductive performance in a facultative colonial breeder.

Barn swallows (*Hirundo rustica*) are a semi-colonial species: individuals breed either in solitary pairs or in colonies of up to dozens of breeding pairs (Brown and Brown 2020). Therefore, this species represents a suitable system to generate ecologically variable comparisons of cognitive traits among conspecific individuals experiencing a varying degree of reproductive sociality. Further, an individual’s degree of sociality during the breeding season appears to have a heritable component, as the group size that an individual is born into is predictive of the group size they eventually settle in as a breeding adult, even when cross-fostered (Brown and Brown 2000; Møller 2002; Roche et al. 2011). Therefore, the social breeding environment that an individual experiences at hatching or during rearing (i.e., natal vs. environmental) has the potential to impact how that same individual responds to cognitively demanding situations.

## Methods

### Study subjects and area

We recorded, tested, and monitored barn swallows that nest in barns and underpasses across Boulder County, Colorado (40ºN, 105ºW), during their breeding season, from May to September 2019. We monitored swallows at 21 sites with colonies ranging from 1 to 33 breeding pairs. Each site consisted of a single breeding pair or colony. Swallows were captured at the beginning of the breeding season and marked with one US Geological Survey aluminum leg band with a unique number, and an additional, unique combination of plastic color bands to allow identification from a distance. Breeding pairs were identified and associated with a nest using periodic focal behavioral observations throughout the breeding season. The contents of their nests were monitored every 3–4 days throughout the entire breeding cycle in order to record hatching dates, the number of eggs hatched, nestling age, and the number of nestlings that survived close to the fledging age (i.e., number of nestlings that were raised to successfully leave the nest).

## Selective attention to social information

### Recordings

We obtained 44 recordings of barn swallow alarm calls in Boulder County across 44 breeding colonies and solitary breeding pairs between May–August 2019. These colonies included the colonies from the 21 sites for which we fully monitored nesting. We elicited alarm calls by approaching active nests (i.e., nests that were being defended by a breeding pair), or by approaching the nest while holding a black-billed magpie (*Pica hudsonia*) decoy attached to a 1 m stick. We recorded swallows with a Marantz PMD 661 digital recorder (Marantz, Chatsworth, CA) in 16-bit WAV format, at a 48 kHz sampling rate, combined with a Sennheiser ME66 shotgun microphone (Sennheiser, Old Lyme, CT).

While recording at barn swallow colonies, we targeted single individuals. However, this was often challenging, because swallows fly in circles around the predator while alarm calling, making individual identification not feasible. Therefore, we selected only one recording per colony for which we were certain that the alarm calls were produced by only one individual.

We obtained 44 mourning dove (*Zenaida macroura*) recordings to use as a control playback stimulus. This is a common and harmless sympatric species whose calls were not expected to elicit antipredator or aggressive responses in barn swallows. These recordings were sourced from two different bioacoustic libraries: www.xeno-canto.org, and www.floridamuseum.ufl.edu/bird-sounds/. Criteria for selecting recordings from the acoustic libraries are stated in the supplementary materials and methods. A full list of the meta-data for these recordings is also provided in the supplementary material, table S1.

*Preparation of playback stimuli*: We generated 30 s-clips of barn swallow alarm calls using Audacity 2.3.3 (www.audacityteam.org). Alarm call recordings were visually inspected to identify a 30 s or shorter section of the recording that contained a continuous rendition of alarm calls, clearly produced by a single individual, without any other alarm calls overlapping. In the case that the rendition found was less than 30 s long, the rendition was copied as many times as needed to complete 30 s. We reproduced the pauses between alarm calls that were observed throughout the recording, in order to obtain a naturalistic 30 s rendition of alarm calls. The same procedure described above was performed to obtain call ‘clips’ of mourning dove calls. Mourning dove calls were also saved in WAV format. Both alarm call and mourning dove clips were normalized to obtain vocalizations with an amplitude of 82 dB at 1 m, which was the measured amplitude of naturally produced barn swallow alarm calls. We used Raven Pro 1.6.0 (ravensoundsoftware.com/software/raven-pro/) to filter frequencies above 2 kHz on mourning dove clips, and below 1 kHz on clips of barn swallow alarm calls to eliminate background noise without eliminating components of the signal (i.e., calls).

*Preparation of playback tracks*: We used Audacity 2.3.3 to assemble playback tracks by pasting together one randomly selected clip of barn swallow alarm calls and one randomly selected clip of mourning dove calls with a period of 2 min of silence between them. Then we repeated those clips five times, alternating the order of both clips. Each repetition of clips represented a trial in the playback experiment. Each trial was separated by either a short (5 min) or a long (10 min) period of silence, which order was alternated. We prepared two types of tracks: one that started with a mourning dove call clip, and a short silence between the first and second trials (supplementary material, figure S1); and the second one started with a swallow alarm call clip and a long silence between the first and second trials. We inserted 30 s of silence before the first clip of each playback track. For each focal bird, we assigned randomly the playback track type that was used for the playback experiment. Each bird was presented with a playback track that contained a unique mourning dove call clip and a unique barn swallow alarm call clip to avoid pseudoreplication (Kroodsma 1989). We ensured that the alarm calls used in each playback experiment were recorded at a different site from where the playback was conducted.

### Playback experiments

The use of social information by incubating adult female barn swallows was tested with playbacks of conspecific alarm calls. These experiments took place between 6 and 11 am. Before starting the experiment, we checked the contents of the focal nest. In instances where it was challenging to distinguish the female from the male at the focal nest, we applied a small amount of non-permanent fluorescent powder (www. bioquipinc.com) on the eggs to mark the incubating female.

We placed a Cambridge SoundWorks OontZ Angle 3XL Ultra Bluetooth Speaker, (www.theoontz.com) speaker 2 m from the nest and set up a hunting blind at least 5 m away from the nest in a location that caused minimal disturbance to the focal nest or any other surrounding nests. Two experimenters entered the blind and allowed for acclimation until the birds resumed their normal activity at the nest. Once the female was sitting on the nest, one experimenter monitored the female’s activity throughout the experiment with binoculars, while the other recorded the setup time, whether predators or humans were present during the experiment, ambient temperature (ºC), and additional notes on the focal bird’s behavior (e.g., whether the focal bird produced alarm calls during the playback experiment).

We used the *Animal Behaviour Pro* Mobile App. Version 1.2 (by Newton-Fisher, N. E.) installed on an Apple iPad (6th generation) to record every time that the female either stood at the nest or left the nest. These behaviors were the most conspicuous and consistent behaviors that females showed in response to conspecific alarm calls. These behaviors also represented two levels of alertness in response to alarm calls, with leaving the nest representing higher alertness than standing at the nest. We recorded these behaviors for 2 min prior to the start of the playback track, during the playback, and 2 min after the playback. If the female left the nest while the playback track was playing, the playback was paused and resumed again once the female returned to the nest, to ensure that the bird was present when the calls were playing.

## Decision-making

Barn swallows exhibit typical anti-predator behavioral responses when researchers approach their nests to check the contents (i.e., leaving the nest, alarm calling, and mobbing) (AMG, personal observation). Thus, we tested decision-making in barn swallows with such simulated predator intrusions at nests that contained nestlings. Similar to some previous studies (Schiavinato et al. 2023; Trnka & Grim 2013), our proxy for decision-making was the latency to return to the nest after a simulated predator intrusion. This latency represents the decision-making process that swallows experience when assessing the risk of returning to their nest to feed their nestlings after a potential predator was at their nest.

We simulated predator intrusions at barn swallow nests between 6 am and 3 pm at an ambient temperature range of 10–30 ºC. We conducted two intrusions at the same nest 1–2 h apart in order to have replicates of the birds’ response to the intrusion (i.e., two trials). Before conducting the intrusion, we ensured that the nest had live nestlings and that both parents at the nest had been identified. For each simulated intrusion, one experimenter intruded the nest while the other performed observations of the focal nest and monitored the latency to return. To avoid pseudoreplication, both experimenters rotated to conduct the intrusions and also wore a different attire at each intrusion. Attires were randomly selected for each focal nest and included unique combinations of shirts, pants, hats, wigs, and sunglasses. Both of the intrusion trials at a given nest were conducted by the same experimenter wearing the same attire.

We noted whether the focal birds flushed or not due to the arrival of the experimenters. We set up a hunting blind at least 5 m from the nest so that the nest was in view, but the blind would not disturb the regular activity of the focal birds or other birds in the colony. The two experimenters entered the blind and allowed for acclimation, until the birds resumed their normal activity around the nest. Once the focal bird, either the male or the female, was at the nest or within a 3 m radius, the experimenter approached the nest at a steady pace and checked its contents. Each intrusion trial lasted 60 s, from the time the experimenter left the hunting blind until it returned to it. All the simulated predator intrusions were recorded with a GoPro HERO4 Session camera (www.GoPro.com) strapped to the intruder’s head. We recorded the setup time, the time at which the focal bird returned to the nest after the intrusion, and additional notes.

### Statistical analyses

Statistical analyses were conducted, and data visualizations created in R version 3.2.1 (R: A Language and Environment for Statistical Computing, R Core Team, R Foundation for Statistical Computing, 2021, Vienna, Austria, https://www.R-project.org).

#### Selective attention to social information

To test the effects of colony size, day into the breeding cycle, predator activity, ambient temperature, and Julian date on the frequency of alert behaviors, we ran generalized linear models (GLM) with a Poisson distribution using the ‘glmmTMB’ package for R (Brooks et al. 2017). We ran one model with the frequency standing at the nest entered as the dependent variable, and another model with the frequency of leaving the nest as the dependent variable. To check for collinearity, or correlations among independent variables that could confound the results of our models, we used Spearman rank correlations to analyze statistical relationships among all possible combinations of our independent variables: colony size, day into the breeding cycle, ambient temperature, and Julian date. None of these variables were highly correlated (*R* < 0.7; supplementary material, Table S2). We further examined relationships among predator activity and the other predictors with Kruskal–Wallis tests (supplementary material, Table S3). We evaluated the fit of each model with the ‘DHARMa’ package for R (Hartig 2021).

To evaluate the link between selective attention to social information and reproductive success we ran generalized linear mixed effects models (GLMM) with a Poisson distribution. Clutch initiation was included as a predictor in all the models because it has been shown to impact reproductive output in barn swallows (Safran 2004, 2006; Safran et al. 2007). Site was added as a random effect in these models. We ran one set of models with the number of eggs hatched as entered as the dependent variable, and another set of models with the number of nestlings that survived to day 12. These represent different measures of seasonal reproductive success with the first incorporating incubation success and the second assessing the total production of offspring for the breeding season. We modeled frequency of standing at the nest and frequency of leaving the nest separately due to the poor model fit when those variables were included in the same model, as evidenced by the residual’s diagnostics with ‘DHARMa’. Therefore, one set of models included the frequency of standing at the nest as a predictor, and the other the frequency of leaving the nest.

#### Decision-making

To test the effects of colony size, simulated intrusion trial (i.e., first and second trial), sex, day into the breeding cycle, and Julian date on the latency to return to the nest, we ran a generalized linear model (GLM) with a Gaussian distribution using the ‘glmmTMB’ package for R. Our model included the log-transformed latency to return to the nest as the dependent variable. We checked for collinearity among the predictor variables included in our models: colony size, day into the breeding cycle, and Julian date, using Spearman correlations. We examined relationships among sex, and the other predictors with Kruskal–Wallis tests. None of the predictors were strongly correlated (supplementary material, Tables S4 and S5).

To examine the link between decision-making and reproductive success we ran generalized linear mixed effects models (GLMM) with a generalized Poisson distribution. Clutch initiation was included as a predictor in all the models and site was added as a random effect. We ran models with the number of nestlings that survived to day 12 entered as the dependent variable. We tested the fit of models including different combinations of trial number, individual ID, and site as random effects, but such models either presented convergence issues or showed a poor fit. The latency to return in the first and the second trial was highly and positively correlated (Spearman rank correlation: *N* = 55, *R* = 0.375, *P* = 0.005), thus both of these variables were not included in the same model. One model included the latency to return in the first trial of the simulated predator intrusion as a predictor, and the other model included the latency to return in the second trial.

## Results

### Selective attention to social information

#### Behavioral responses to the playback of conspecific alarm calls

When comparing the average frequency of standing on the nest across the stages of the playback experiment (i.e., pre-playback: no sounds played, alarm call playback, control: mourning dove call, and post-playback: no sounds played), we found that the frequency of this behavioral response was highest during the playback of conspecific alarm calls (GLM, estimate = 0.625, sd = 0.212, *P* = 0.003; supplementary material, figure S2a). We found the same for the frequency of leaving the nest (GLM, estimate = 0.859, sd = 0.203, *P* < 0.001; supplementary material, figure S2b).

#### Consistency in female behavioral responses to the playback of conspecific alarm calls

A prerequisite for analyzing how females respond to various experimental treatments is that these behaviors are consistent within individuals. To establish this, we calculated consistency scores for the two behavioral responses that we observed during our playback of conspecific alarm calls: standing at the nest and leaving the nest (supplementary material, figure S3). The consistency score we used was the proportion of trials in which a female exhibited one of the alert behaviors (i.e., standing at the nest or leaving the nest). In four randomly chosen trials out of five total, females displayed consistent responses to the experimental treatment. Specifically, whether females stood or not at the nest, or left the nest or not, they were consistent in their response across these trials (all of them showed a score larger than 0.5). Due to this consistency, we included only 4 randomly selected trials in the analyses to have a larger sample size (44 individuals with 4 completed trials instead of only 35 females that completed all 5 trials).

#### Correlation between behavioral responses to the playback of conspecific alarm calls

Finally, we analyzed whether the behavioral responses to the playback were correlated and found that they were not (Spearman rank correlation: *N* = 44, *R* = 0.152, *P* = 0.323). We, thus, infer these two behaviors (standing and leaving the nest) are two distinct behavioral responses to conspecific alarm calls.

#### Behavioral responses to the playback of conspecific alarm calls

**Fig. 1:**
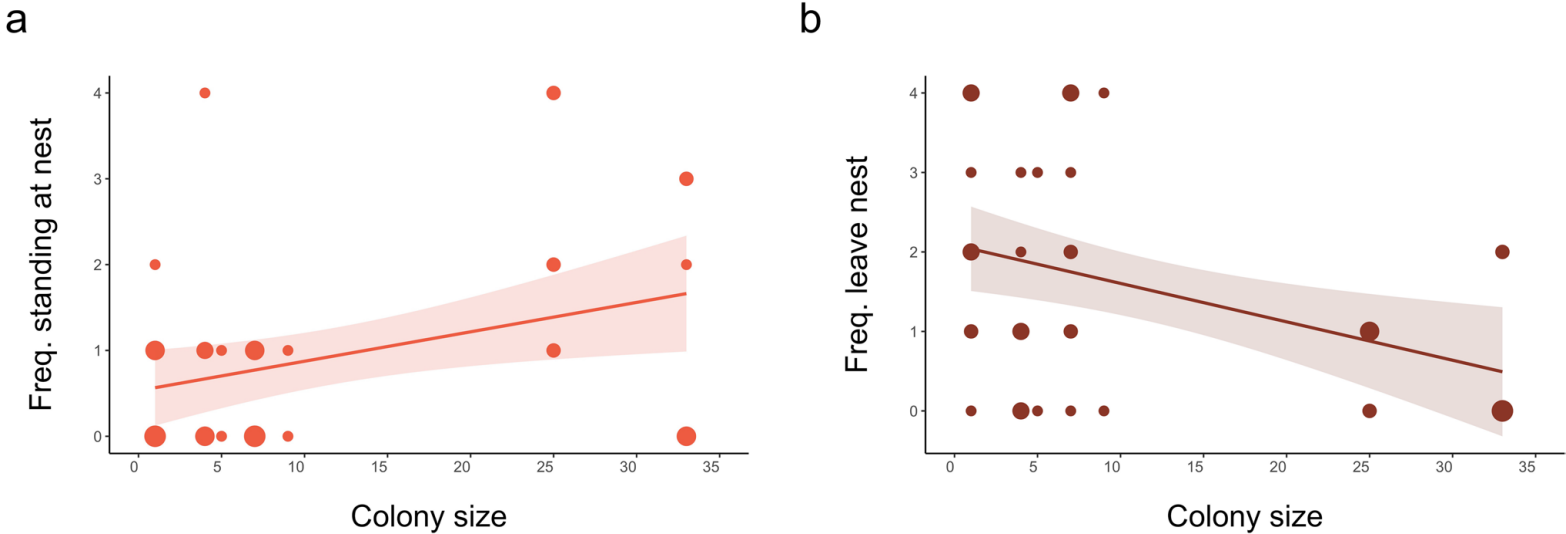
**a** Relationship between colony size and frequency of standing at the nest during eight minutes of playback of conspecific alarm calls (*N* = 44). **b** Relationship between colony size and frequency of leaving the nest during playback of conspecific alarm calls (*N* = 44). The size of points represents the number of overlapping points. Shaded areas represent 95% confidence intervals from the generalized linear models

Parents from large colonies stood at the nest significantly more often and left the nest less often than birds from smaller colonies in response to an alarm call (Table 1, Fig. 1). Our results support the prediction that individuals in larger colonies will be more selective in their acquisition of social information (i.e., less responsive to conspecific alarm calls) than individuals breeding in smaller colonies or solitarily.

**Table 1:** Effects of colony size, day into the breeding cycle, predator activity, ambient temperature, and Julian date on alert behaviors (frequency of standing at the nest and leaving the nest)

Response variable	Explanatory variables	Estimate (mean ± SE)	*Z* value	*P* value
Standing at nest	Intercept	− 0.167 ± 2.732	− 0.061	0.951
	**Colony size**	**0.040 ± 0.014**	**2.863**	**0.004**
	Day into breeding cycle	0.033 ± 0.064	0.519	0.604
	Predator activity	− 0.500 ± 0.360	− 1.386	0.166
	Ambient temperature	0.049 ± 0.038	1.293	0.196
	Julian date	− 0.008 ± 0.011	− 0.723	0.470
Leaving nest	Intercept	0.553 ± 1.837	0.301	0.764
	**Colony size**	− **0.041 ± 0.015**	− **2.819**	**0.005**
	Day into breeding cycle	0.073 ± 0.045	1.625	0.104
	Predator activity	0.146 ± 0.271	0.539	0.590
	Ambient temperature	− 0.005 ± 0.026	− 0.182	0.855
	Julian date	− 0.004 ± 0.008	− 0.59	0.555

Significant results are indicated in bold. *N* = 44

### Decision-making

**Fig. 2:**
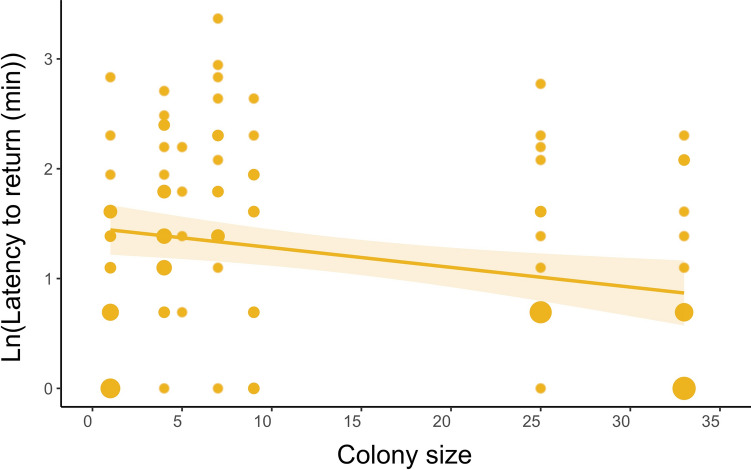
Relationship between colony size and the log-transformed latency to return to the nest after the simulated predator intrusion (*N* = 55; two trials per bird). The size of points represents the number of overlapping points. The shaded area represents 95% confidence intervals from the generalized linear model

Our results support the prediction that individuals in larger colonies will return to the nest more quickly (i.e., show shorter latency to return to the nest) after a predator intrusion than individuals breeding in smaller colonies or solitarily (Table 2, Fig. 2). On average, birds from our largest colony returned to the nest 1.5 times faster than birds from solitary sites.

**Table 2:** Effects of colony size, trial, sex, day into the breeding cycle, and Julian date on latency to return to the nest after the simulated predator intrusion

Explanatory variables	Estimate (mean ± SE)	*t* value	*P* value
Intercept	1.700 ± 1.691	1.005	0.317
**Colony size**	**− 0.017 ± 0.007**	**− 2.533**	**0.013**
Trial	**− **0.002 ± 0.165	**− **0.009	0.993
Sex	**− **0.024 ± 0.169	**− **0.1401	0.889
Day into breeding cycle	**− **0.010 ± 0.018	**− **0.525	0.600
Julian date	2.28 × 10^–4^ ± 0.007	0.031	0.975

Significant results are indicated in bold

### Reproductive outcomes associated with selective attention and decision-making

**Fig. 3:**
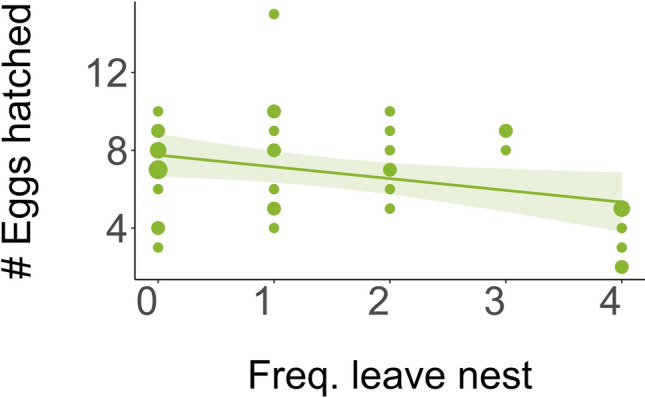
Relationship between total number of eggs hatched and the frequency to leave the nest during eight minutes of playback of conspecific alarm calls (*N* = 41). The size of points represents the number of overlapping points. The shaded area represents 95% confidence intervals from the generalized linear model without site

**Fig. 4:**
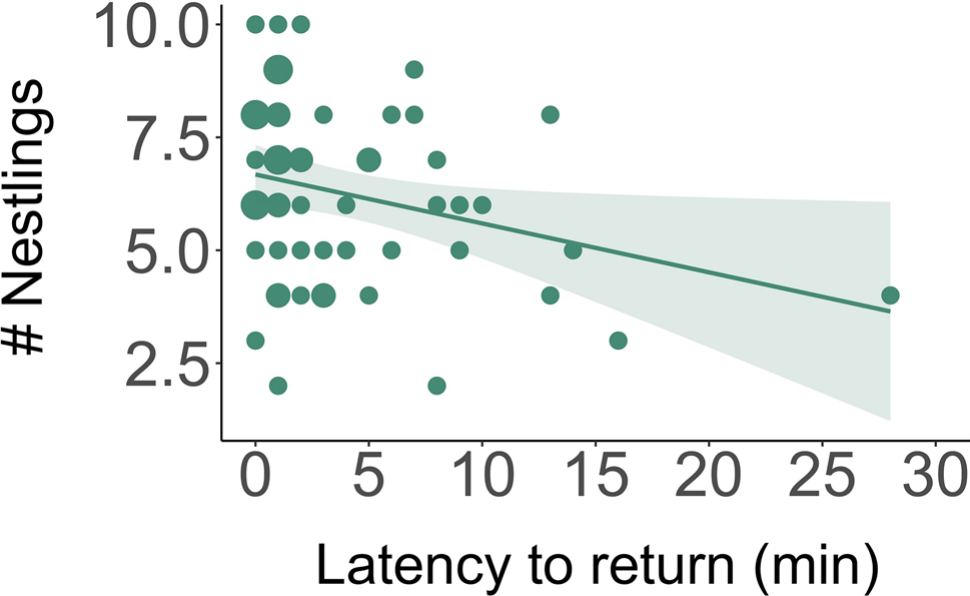
Relationship between total number of nestlings that survived until day 12 and the latency to return to the nest after a simulated predator intrusion (*N* = 41). The size of points represents the number of overlapping points. The shaded area represents 95% confidence intervals from the generalized linear model without site

Here, we report several measures of reproductive outcomes in association with measures of selective attention (Table 3, Fig. 3) and decision-making (Table 4 and Fig. 4) using two relevant measures of reproductive success: total number of eggs hatched and total number of nestlings that survived until leaving the nest.

**Table 3:** Effects of alert behaviors (frequency of standing at the nest and leaving the nest), site and clutch initiation on 1) the total number of eggs hatched, and 2) total number of nestlings that survived until day 12

Response variable	Effect	Estimate (mean ± SE)/Variance (mean ± SD)	*Z* value	*P* value
Total number of eggs hatched	**Fixed**			
	Intercept	2.782 ± 0.881	3.158	0.002
	Standing at nest	0.053 ± 0.054	0.977	0.329
	Clutch initiation	− 0.006 ± 0.006	− 1.022	0.307
	**Random**			
	Site	2.45 × 10^–10.^ ± 1.65 × 10^–5^		
Total number of nestlings	**Fixed**			
	Intercept	2.584 ± 0.938	2.755	0.006
	Standing at nest	0.079 ± 0.052	1.52	0.128
	Clutch initiation	− 0.006 ± 0.006	− 0.952	0.341
	**Random**			
	Site	2.30 × 10^–10.^ ± 1.52 × 10^–5^		
Total number of eggs hatched	**Fixed**			
	Intercept	2.758 ± 0.895	3.082	0.002
	**Leaving nest**	**− 0.091 ± 0.043**	− **2.12**	**0.034**
	Clutch initiation	− 0.005 ± 0.006	− 0.791	0.429
	**Random**			
	Site	1.05 × 10^–10.^ ± 1.02 × 10^–5^		
Total number of nestlings	**Fixed**			
	Intercept	2.523 ± 0.966	2.612	0.009
	Leaving nest	− 0.067 ± 0.045	− 1.495	0.135
	Clutch initiation	− 0.004 ± 0.006	− 0.686	0.493
	**Random**			
	Site	3.90 × 10^–10.^ ± 1.97 × 10^–5^		

Significant results are indicated in bold. *N* = 44

**Table 4:** Effects of the latency to return to the nest after a simulated predator intrusion on the total number of nestlings that survived until day 12

Effect	Estimate (mean ± SE)/Variance (mean ± SD)	*Z* value	*P* value
Fixed			
Intercept	3.283 ± 0.801	4.098	4.17 × 10^–5^
Latency to return to nest (Trial 1)	**− **0.006 ± 0.010	**− **0.602	0.547
Clutch initiation	**− **0.009 ± 0.005	**− **1.836	0.066
Random			
Site	0.014 ± 0.117		
Fixed			
Intercept	3.462 ± 0.826	4.192	2.77 × 10^–5^
Latency to return to nest (Trial 2)	**− 0.021 ± 0.009**	**− 2.316**	**0.021**
Clutch initiation	**− 0.010 ± 0.005**	**− 1.905**	**0.057**
Random			
Site	0.001 ± 0.036		

Results are shown separately for the first and the second trial of the simulated predator intrusions. Estimates and SE are given for fixed effects and variance and SD are given for random effects. Significant results are indicated in bold

#### Selective attention to social information

Controlling for clutch initiation date, we found a significant negative relationship between a single measure of reproductive success (the number of eggs hatched) and the frequency of leaving the nest, indicating that individuals who left their nest least often hatched more eggs in their nest compared to others who left more often (Table 3, Fig. 3).

#### Decision-making

Controlling for clutch initiation date, we found a significant negative relationship between a single measure of reproductive success (the estimated number of nestlings fledged) and the latency to return to the nest on the second trial of the simulated predator intrusion, indicating that individuals who stayed away from their nests for a shorter time period after the simulated nest intrusion had more nestlings survive (Table 4, Fig. 4).

## Discussion

We experimentally tested two fundamental predictions of the social intelligence hypothesis. First, we analyzed whether individuals in larger colonies are more selective in their attention to social information (i.e., less responsive to conspecific alarm calls by being more likely to just stand at their nest instead of leaving it during the playback of a conspecific alarm call) than individuals breeding in smaller colonies or solitarily. Our results show that individuals breeding in larger colonies are less responsive to conspecific alarm calls than solitary breeders, which suggests that breeders at larger colonies are more selective in their attention to social information than solitary ones, supporting a key prediction of the social intelligence hypothesis. Next, we analyzed whether individuals in larger colonies decide to return to the nest more quickly after a simulated predator intrusion. Our results indicate that birds breeding in larger colonies returned more quickly, suggesting that decision-making processes in this species are influenced by their social environment. Finally, we analyzed whether these colony-size dependent cognitive traits are also associated with measures of seasonal reproductive success. We found that birds that show higher selective attention (i.e., were less likely to leave their nest in response to alarm calls), hatched more eggs. Furthermore, birds that decided to return to their nest more quickly after a simulated predator intrusion had more nestlings, supporting the impact of decision-making processes on a fitness component (i.e., seasonal reproductive success) in this species. Taken together, our results suggest that social breeding plays a role in shaping the acquisition of social information and decision-making in barn swallows.

Critically, these results also link colony-size dependent cognitive traits with metrics of reproductive success in the same individuals: both the number of eggs hatched, and the number of nestlings produced were positively related to a proxy for cognitive performance, namely the inverse of both the frequency of departing the nest and the latency of returning to the nest after departure.

Our results indicate that individuals in larger colonies were more selective in their use of social information than individuals in smaller colonies. Individuals from large colonies also showed shorter latency to return to the nest after a predator intrusion, which suggests that individuals in larger colonies may have reduced risk avoidance, as shown previously in other species (Whiteside et al. 2016). This selective attention and reduced risk avoidance is consistent with our prediction that colony size affects individuals’ use of social information and decision-making. One explanation for these results is that individuals in larger groups have the added benefit of social information for decision-making, while solitary pairs must base decisions solely on individually acquired information. With multiple sources of available information, individuals in larger groups may exploit the most reliable source when making decisions (van Bergen et al. 2004). However, another explanation for the observed selective attention could be the dilution of predatory risk with larger group size as defined in the ‘many eyes’ hypothesis, where individuals can scan for predators less frequently without losing predator detection (Lima 1995; Roberts 1996).

Though strictly correlational in nature, we show that female barn swallows that were less likely to leave their nest in response to alarm calls hatched more eggs, which suggests that females that are not selective in their attention to social cues incur a fitness cost. Similarly, birds that show higher risk avoidance by taking longer to return to their nests after the simulated predator intrusion appear to incur a fitness cost by producing fewer nestlings. We recognize that given our inability to randomize, control, or measure diverse other socio-ecological factors that may affect fitness between different colony sizes, we cannot fully attribute these results to the cognitive measures considered in this study. Alternatively, our results may be interpreted as a differential parental investment according to brood size; that is, swallows with larger broods are less likely to leave their nest during playbacks of alarm calls and return to the nest more quickly after a simulated predator intrusion, due to the higher value of large broods. Although we cannot rule out this alternative explanation with our current data, it is clear that such adjustments in antipredator behaviors according to brood size would require underpinning cognitive processes, such as information processing, and the subsequent use of social information as well as decision-making.

Overall, our results are consistent with the few existing intraspecific appraisals of the social intelligence hypothesis (Ashton et al. 2018a, b; Langley et al. 2018; Liker and Bokony 2009). Group size has been shown to play an important role on cognitive traits in Australian magpies (*Cracticus tibicen dorsalis*), and more importantly, these cognitive traits have also been shown to be associated to multiple measures of reproductive success (Ashton et al. 2018a, b). In contrast to other studies, we evaluated cognitive traits that can be directly linked to their adaptive value to social living, for instance, selective attention to social information is a trait that is highly relevant in social settings. Such traits are the ‘building blocks’ of social cognition (Seyfarth and Cheney 2015). Our study further adds to the body of evidence that supports the role of social living on the evolution of cognition in nonhuman vertebrates.

We neither manipulated individuals’ experience across the different colony sizes, nor did we track cognitive, physiological, or neural responses to the experimental stimuli presented in our trials. Thus, it remains unclear what aspects of individuals’ perception, neural processing, or decision responsiveness were dependent on colony size in our experimental subjects (Mendelson et al. 2016). We also found substantial intraindividual consistency and interindividual variation in selective attention and decision-making, which warrants further investigation. Among many factors influencing this variation, it is possible that individuals base their behavioral responses by copying the responses of fellow colony members nearby, a possibility that should be explored in future studies. To gain a deeper understanding of individual variation, future research should examine genetic and environmental components of variation in cognitive traits across breeding and other types of social groups’ sizes.

## Supplementary Information

Below is the link to the electronic supplementary material.

Supplementary file1 (DOCX 3024 KB)

## Data Availability

Data supporting this study can be accessed through Figshare using the link: https://figshare.com/s/c8ccba2293d60a0455e6
