# Supplementary material for: Colonial breeding impacts potentially fitness-relevant cognitive processes in barn swallows
Source: Anim Cogn. 2024 Mar 2;27(1):15. doi: 10.1007/s10071-024-01841-1 (PMC10907464; doi:10.1007/s10071-024-01841-1)
Supplement: Supplementary file 1 — Supplementary file1 (DOCX 3024 KB) [file 10071_2024_1841_MOESM1_ESM.docx]

**Social breeding impacts fitness-relevant cognitive processes in barn swallows**

Angela Medina-García, Ellen Scherner, Molly T. McDermott, Mark E. Hauber, and Rebecca J. Safran

**Supplementary Material**

**Supplementary Methods**

***Playback file selection from bioacoustic libraries***

When selecting recordings from both libraries, we avoided recordings that contained songs from other species overlapping the mourning dove vocalizations. We only selected recordings from Xeno-canto.org that were rated as ‘A’ quality, with a bit rate of mp3 equal or higher than 128,000 bps. We only selected mourning dove calls whose spectrograms looked similar to the most commonly found call type in this species in North America.

Table S1. Source, catalogue number, date, recordist, and country of mourning dove (*Zenaida macroura*) call recordings used in the study.

| Source | Catalog # | Date | Recordist | Country |
| --- | --- | --- | --- | --- |
| 1 | FLMNH04371 | 1978-09-03 | J.W. Hardy | United States |
| 1 | FLMNH05220 | 1980-04-19 | J.W. Hardy | United States |
| 1 | FLMNH10267 | 1978-01-01 | Andres M. Sada | Mexico |
| 1 | FLMNH13021 | 1988-08-07 | Andres M. Sada | Mexico |
| 1 | FLMNH22292 | 1964-07-19 | Ben B, Jr. Coffey | United States |
| 1 | FLMNH22674 | 1964-07-19 | Ben B, Jr. Coffey | United States |
| 1 | FLMNH28048 | Unknown | Unknown | United States |
| 1 | FLMNH30536 | 2019-05 | Unknown | United States |
| 2 | XC109033 | 2012-05-22 | Andrew Spencer | United States |
| 2 | XC131448 | 2012-06-29 | Eric DeFonso | United States |
| 2 | XC14871 | 2007-04-21 | Andrew Spencer | United States |
| 2 | XC14872 | 2007-05-25 | Andrew Spencer | United States |
| 2 | XC153639 | 2013-04-21 | Paul Marvin | United States |
| 2 | XC153651 | 2012-05-21 | Paul Marvin | United States |
| 2 | XC153652 | 2012-06-07 | Paul Marvin | United States |
| 2 | XC164123 | 2013-07-09 | Patrick Turgeon | Canada |
| 2 | XC179721 | 2014-05-24 | Martin St-Michel | Canada |
| 2 | XC196881 | 2014-08-25 | Dan Lane | United States |
| 2 | XC232557 | 2007-02-18 | Peter Boesman | United States |
| 2 | XC253387 | 2015-05-31 | Eric DeFonso | United States |
| 2 | XC265409 | 2015-08-02 | Manuel Grosselet | Mexico |
| 2 | XC268200 | 2015-05-17 | Richard E. Webster | United States |
| 2 | XC268205 | 2015-05-19 | Richard E. Webster | United States |
| 2 | XC268206 | 2015-05-17 | Richard E. Webster | United States |
| 2 | XC268207 | 2015-05-25 | Richard E. Webster | United States |
| 2 | XC268208 | 2015-06-16 | Richard E. Webster | United States |
| 2 | XC268209 | 2015-05-29 | Richard E. Webster | United States |
| 2 | XC268211 | 2015-05-25 | Richard E. Webster | United States |
| 2 | XC268212 | 2015-05-05 | Richard E. Webster | United States |
| 2 | XC286962 | 2015-05-03 | Paul Marvin | United States |
| 2 | XC309695 | 2016-03-29 | Manuel Grosselet | Mexico |
| 2 | XC316787 | 2016-04-29 | Thomas G. Graves | United States |
| 2 | XC329214 | 2016-07-29 | Antonio Xeira | United States |
| 2 | XC35102 | 2009-05-29 | Andrew Spencer | United States |
| 2 | XC354053 | 2017-02-02 | Lance A. M. Benner | United States |
| 2 | XC368461 | 2017-04-26 | Dan Lane | United States |
| 2 | XC381087 | 2017-07-17 | Manuel Grosselet | Mexico |
| 2 | XC381111 | 2017-07-16 | Manuel Grosselet | Mexico |
| 2 | XC381112 | 2017-07-16 | Manuel Grosselet | Mexico |
| 2 | XC407859 | 2011-06-20 | Jim Berry | United States |
| 2 | XC412632 | 2018-03-31 | Dan Minor | Canada |
| 2 | XC440853 | 2018-07-18 | Richard E. Webster | Mexico |
| 2 | XC451819 | 2018-03-19 | Paul Marvin | United States |
| 2 | XC451916 | 2018-06-25 | Paul Marvin | United States |
| 2 | XC46669 | 2010-04-05 | Daniel Lane | Puerto Rico |

1- Bird sound collection of the Florida Museum of Natural History

2- www.xeno-canto.org

Table S2. Spearman rank correlations among colony size, day into the breeding cycle, ambient temperature, and Julian date associated to the playback experiments.

|  | Colony size | Day into breeding cycle | Ambient temperature | Julian date |
| --- | --- | --- | --- | --- |
| Colony size | 1.000 | 0.096 | -0.019 | 0.167 |
| Day into breeding cycle | 0.096 | 1.000 | -0.138 | -0.229 |
| Ambient temperature | -0.019 | -0.138 | 1.000 | 0.083 |
| Julian date | 0.167 | -0.229 | 0.083 | 1.000 |

Table S3. Kruskal-Wallis tests between predator activity during playback experiments and colony size, day into breeding cycle, ambient temperature, and Julian date. Chi-squared values (χ2**)**, degrees of freedom (df), and P-values are shown.

| Variable | χ2 | df | *P-value* |
| --- | --- | --- | --- |
| Colony size | 0.079 | 1 | 0.779 |
| Day into breeding cycle | 3.764 | 1 | 0.052 |
| Ambient temperature | 0.468 | 1 | 0.494 |
| Julian date | 0.824 | 1 | 0.364 |

Table S4. Spearman rank correlations among colony size, day into the breeding cycle, and Julian date associated to the playback experiments.

|  | Colony size | Day into breeding cycle | Julian date |
| --- | --- | --- | --- |
| Colony size | 1.000 | 0.400 | 0.038 |
| Day into breeding cycle | 0.400 | 1.000 | -0.108 |
| Julian date | 0.038 | -0.108 | 1.000 |

Table S5. Kruskal-Wallis tests between sex and colony size, day into breeding cycle, and Julian date during the simulated predator intrusions. Chi-squared values (χ2**)**, degrees of freedom (df), and P-values are shown.

| Variable | χ2 | df | *P-value* |
| --- | --- | --- | --- |
| Colony size | 0.161 | 1 | 0.688 |
| Day into breeding cycle | 3.839 | 1 | 0.050 |
| Julian date | 0.665 | 1 | 0.415 |


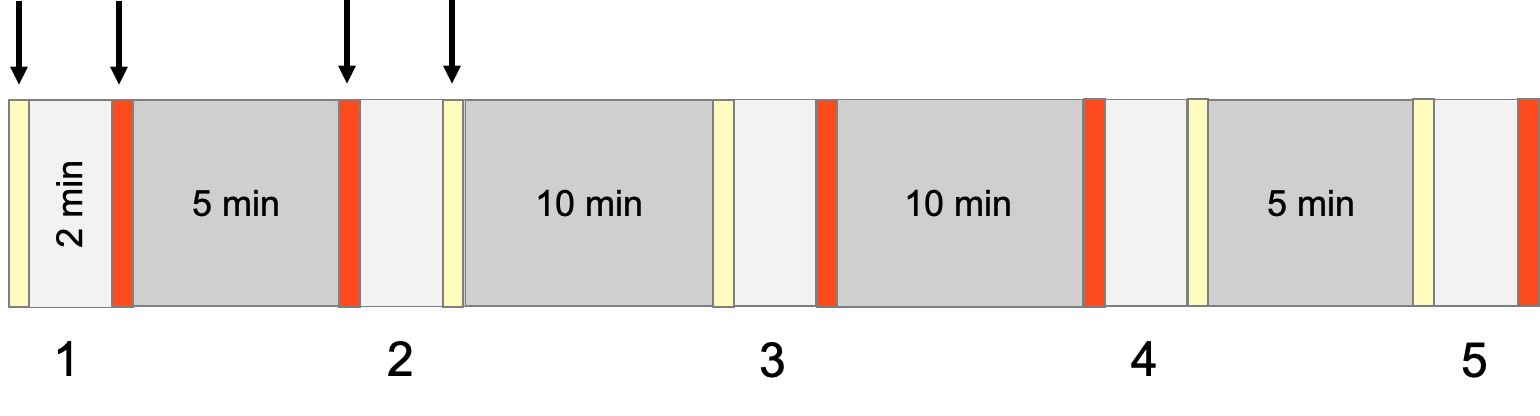


Figure S1. Example of one type of playback track presented to female barn swallows in this study. Light yellow bars represent 30s-clips of mourning dove calls and red bars represent 30s-clips of barn swallow alarm calls. Grey rectangles represent periods of silence. Numbers 1–5 indicate the trial number in the playback experiment.


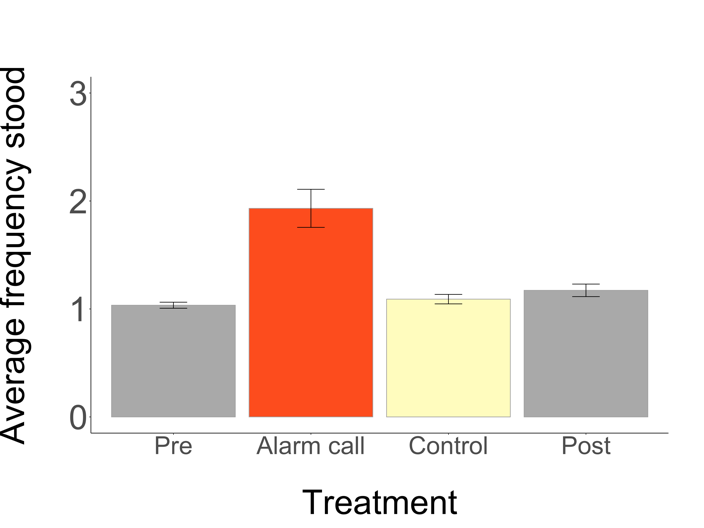
a b


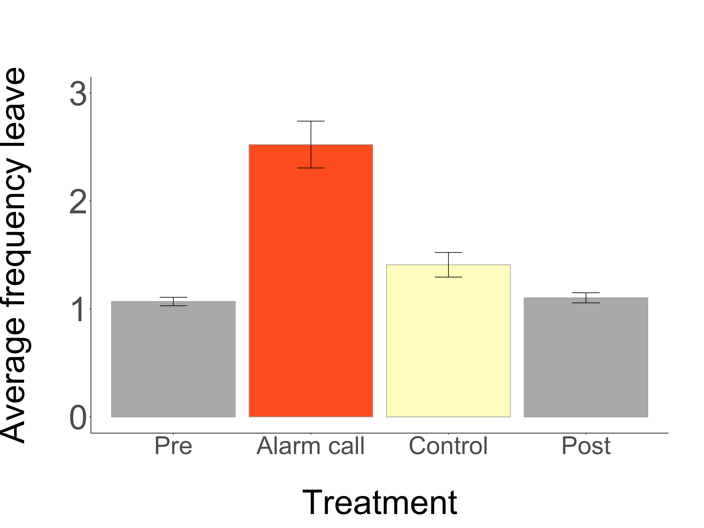


Figure S2. Comparison of alert behaviors (a: standing at the nest, b: leaving the nest) shown by female barn swallows across playback treatments (pre and post playback, barn swallow alarm calls, mourning dove vocalizations). Mean values and SEM are given.


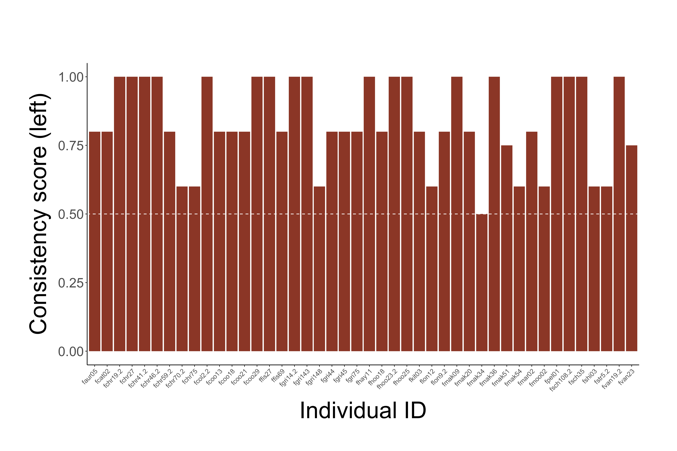

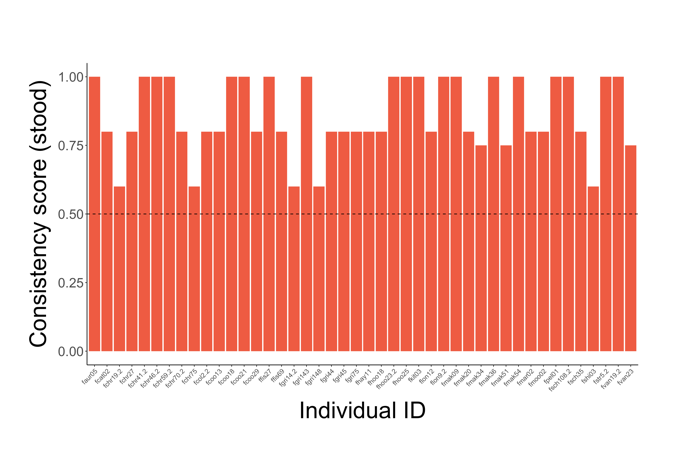
a b

Figure S3. Consistency score of alert behaviors (a: standing at the nest, b: leaving the nest) shown by female barn swallows in four out five trials. The four trials for each female were randomly selected.
